# Déterminants de l'inutilisation de la contraception moderne chez les femmes en âge de procréation dans une zone post-conflit: cas de la Zone de santé d'Ibanda, ville de Bukavu, Est de la République démocratique du Congo

**DOI:** 10.11604/pamj.2026.53.153.21571

**Published:** 2026-04-13

**Authors:** Sylvie Nabintu Mwambali, Théophile Mitima Kashosi, Jean Paul Cikwanine, Mukanire Ntakwinja, Olivier Nyakio, Denis Mukwege

**Affiliations:** 1 Département de Gynéco-obstétrique, Hôpital de Panzi, Faculté de Médecine, Université Evangélique en Afrique, Bukavu, République démocratique du Congo,; 2 Département des Sciences Biomédicales, Faculté de Médecine, Université Evangélique en Afrique, Bukavu, République démocratique du Congo,; 3 Département de Chirurgie, Hôpital de Panzi, Faculté de Médecine, Université Evangélique en Afrique, Bukavu, République démocratique du Congo,; 4 Fondation Panzi, Bukavu, République démocratique du Congo

**Keywords:** Contraception, inutilisation, zone à conflit, Contraception, non-use, conflict zone

## Abstract

**Introduction:**

l'objectif de cette étude était de déterminer les facteurs prépondérants à l'inutilisation des méthodes contraceptives modernes chez les femmes en âge de procréer, de la Zone de santé d'Ibanda.

**Méthodes:**

du 1^er^ au 31 mai 2019, nous avons réalisé une étude descriptive transversale. Les femmes âgées de 13 à 45 ans, non enceintes, habitant la Zone de santé d'Ibanda depuis deux ans nous ont intéressées. La collecte des données a été réalisée par une interview libre. La prévalence contraceptive moderne se référait aux femmes utilisant les contraceptifs modernes au moment de l'enquête. Les données avaient été analysées grâce au logiciel IBM SPSS version 20. La comparaison des proportions a été réalisée au seuil de signification de 5%, avec un rapport de cote supérieur à 1 et l'intervalle de confiance excluant 1 à sa borne inférieure.

**Résultats:**

la prévalence d'utilisation des méthodes contraceptives modernes était de 23,9%, plusieurs femmes (67,2%) refusaient d'utiliser les méthodes contraceptives modernes malgré l'information dont elles disposaient, à cause de leur désir de maternité, le fait d'avoir perdu un enfant en couche (OR: 0,07; IC95%: 0,03-0,18; p=0,000).

**Conclusion:**

l'amélioration du taux de survie des enfants pour chaque ménage, indicateur clé de la qualité des soins obstétriques et pédiatriques d'un pays, et la meilleure sensibilisation face à la désinformation sur les risques d'infertilité après usage des méthodes contraceptives, sont le préalable pour accroître l'utilisation des méthodes contraceptives modernes dans la Zone de santé d'Ibanda.

## Abstract (en)

**Introduction:**

the purpose of this study was to determine predominant factors for modern contraceptive non-use in women of childbearing age living in the Ibanda Health Zone.

**Methods:**

we conducted a descriptive cross-sectional study from May 1 to May 31, 2019. All women aged 13-45 who were not pregnant and had been living in the Ibanda Health Zone for two years, were enrolled. Data collection was carried out by free interview. Modern contraceptive prevalence referred to women using modern contraceptives at the time of the survey. Data were analyzed using IBM SPSS 20 software. Proportions were compared setting significance threshold at 5%, with an odds ratio greater than 1 and a confidence interval excluding 1 at its lower bound.

**Results:**

the prevalence of modern contraceptive utilization was 23,9%, several women (67,2%) refused to use modern contraceptive methods, despite the information they had, because of their desire for maternity and the loss of a baby during delivery (OR: 0.07; CI95%: 0.03-0.18; p=0.000).

**Conclusion:**

improving the survival rate for children in each household, a key indicator for the quality of obstetric and pediatric care in a country and increasing awareness on disinformation about the risks of infertility by contraceptive use are prerequisites to increase the use of modern contraceptive methods in the Ibanda Health Zone.

## Introduction

La contraception, qui a pour but d'éviter une grossesse, est définie comme l'ensemble des moyens employés pour provoquer une infécondité temporaire chez la femme ou chez l'homme [1]. Les grossesses trop précoces, trop tardives, trop nombreuses et trop rapprochées sont responsables de la majorité des complications obstétricales directes, causes de plus de 70% des décès maternels dans les pays à faible revenu [2,3]. Au niveau mondial, l'utilisation des contraceptifs a augmenté, passant de 54% en 1990 à 57,4% en 2014. Ceci s'est vu en particulier en Asie et en Amérique latine, mais reste faible en Afrique subsaharienne. Pourtant, on estime à 225 millions le nombre de femmes qui souhaiteraient éviter ou espacer les grossesses dans les pays en développement, mais qui n'utilisent aucune méthode de contraception, à cause d'un manque de choix, d'un accès limité, de la crainte d'effets secondaires, d'opposition culturelle ou religieuse [4]. Des travaux scientifiques ont montré que l'âge, la religion, le niveau d'instruction, l'autorisation du conjoint et le lieu de résidence avaient une influence sur l'utilisation de la contraception moderne [5,6].

En République démocratique du Congo (RDC), le profil sanitaire par rapport aux décès maternels reste alarmant, une enquête de 2007-2014 conclut à 846 décès pour 100 000 naissances vivantes [6], et en 2017, 473 décès pour 100 000 naissances vivantes, une baisse avait été notée en termes de mortalité maternelle corrélée à une amélioration du taux d'utilisation de la contraception qui de 2007 à 2015, était passé de 13 à 20% [7]. Depuis 1994, la province du Sud-Kivu en RDC est en proie à une situation de guerre et de conflits armés. Cette situation a entraîné des conséquences dramatiques dans la vie de la population de cette province: des millions de morts, des réfugiés et des déplacés, des infrastructures de base détruites ou endommagées. Plus la taille du ménage est élevée, plus celui-ci est exposé à la pauvreté et vice-versa. Il existe une corrélation entre pauvreté et prévalence contraceptive (PC) [8]. Dans le cadre de s'aligner sur les objectifs du plan de développement durable dans la réduction de la mortalité maternelle et infantile et la surveillance des décès maternels et riposte (SDMR), 18 DPS sur 26 DPS ont notifié plus de 100 décès maternels (DM) pour le premier semestre de 2018, dont le Sud-Kivu en tête avec un total de 361 cas de DM avec une prévalence contraceptive encore faible [9,10]. Cette situation alarmante dans cette province de la RDC motive cette étude qui vise à déterminer les facteurs favorisants l'inutilisation de la contraception moderne chez les femmes en âge de procréation.

## Méthodes

**Type d'étude:** cette étude du type transversal à visée analytique réalisée dans la Zone de santé d'Ibanda, ville de Bukavu, Province du Sud-Kivu au second semestre de l'année 2019.

**Site d'étude:** la Zone de santé d'Ibanda est l'une de 34 zones de santé que compte la province du Sud-Kivu. Elle est identifiée par le code 06010202, subdivisée en 16 aires de santé pour une population totale de 452608 habitants avec une taille de ménage moyenne de 5,5 et 82292 ménages au total. La population des femmes en âge de procréer représente 15% de la population totale dans la zone urbaine [7]. La population des adolescentes (10-24 ans) représente 32,8% de la population totale, soit une population de 95048 femmes en âge de procréer [7]. Parmi les 16 aires de santé de la Zone de santé, 15 sont couvertes par un centre de santé et 1 par un centre hospitalier.

**Population d'étude:** notre population d'étude était constituée essentiellement de femmes en âge de procréer.

**Critères d'inclusion:** nous avons inclus dans notre étude toute femme âgée de 13 à 45 ans, résidant dans la Zone de santé d'Ibanda pendant au moins une année, présente dans le ménage le jour de l'enquête et qui aurait accepté de participer volontairement à l'étude.

**Echantillonnage:** nous avons calculé la taille de l'échantillon en utilisant la formule générale de Schwartz [10]: N = Zα^2^.p.q.e /d^2^

Où Zα = degré de confiance à 95% (1,96); p = taux des femmes en âge de procréer (15%); q = 1 - p = 1 - 0,15 = 0,85 ; e = effet de grappe (2); d = précision souhaitée (5%). Ainsi, N = (1,96)^2^ x 0,15 x 0,85 x 2 / (0,05)^2^, n = 391,8 = 392. Les non-répondants sont estimés à 10% de la taille de l'échantillon. Ce qui revient à dire que N = 431. Pour le choix de l'échantillon, nous avons utilisé la technique d'échantillonnage probabiliste du type échantillonnage stratifié pondéré.

Les 16 aires de la Zone de santé d'Ibanda étaient considérées comme des strates. Dans chaque strate (aire de santé), un échantillon proportionnel à la taille de la population de l'aire de santé était tiré de manière aléatoire. Ainsi, l'enquêteur se plaçait au milieu de l'aire de santé concernée et jetait une bouteille en plastique en l'air et la laissait tomber par terre. La direction de l'ouverture de la bouteille était celle que prenait l'enquêteur. Ce dernier entrait dans la première maison (1^er^ ménage) pointée par le goulot de la bouteille; là, l'enquêteur expliquait le but de l'étude pour obtenir le consentement et après le questionnaire était lu à la femme trouvée et cette dernière répondait librement. Les autres ménages suivant le premier étaient enquêtés jusqu'à obtenir la taille voulue de l'échantillon dans l'aire de santé. Au cas où dans le ménage sélectionné il n'y a pas de femme en âge de procréer, l'enquêteur entrait dans le ménage suivant. L'enquête s'est déroulée entre le 1^er^ et le 31 mai 2019. Quatre cent deux (402) enquêtées ont répondu favorablement et complètement à nos questions.

### Variables de l'étude

***Variables dépendantes:*** utilisation des méthodes contraceptives par les femmes en âge de procréer.

***Variables indépendantes:*** caractéristiques sociodémographiques des femmes, variables liées à la connaissance sur la contraception et attitudes des femmes sur la contraception.

### Analyse statistique des données

***Analyse descriptive:*** pour décrire nos enquêtées, nous avons utilisé les effectifs et le pourcentage pour chaque variable catégorielle tandis que la moyenne et l'écart type étaient utilisés pour les variables quantitatives.

***Analyse bivariée:*** nous avons cherché l'association entre la variable dépendante (utilisation des méthodes contraceptives modernes) et les variables indépendantes (connaissances et l'attitude des femmes sur la contraception) pour évaluer si ces dernières étaient associées de manière significative. Ainsi le rapport de cotes (OR), son intervalle de confiance à 95% (IC 95%) et la valeur p nous indiquaient si oui ou non, il y avait association entre la variable dépendante et la variable indépendante. Nous avons considéré qu'il y avait une association lorsque OR était supérieur à 1, IC 95% excluant 1 à sa borne inférieure et la valeur de p inférieure à 0,05.

***Analyse multivariée:*** nous avons essayé d'associer toutes les variables qui étaient significativement associées à l'utilisation de la contraception dans l'analyse bivariée pour éliminer les facteurs de confusion. Les mêmes critères de l'analyse bivariée étaient appliqués pour affirmer qu'il y a association significative entre les différentes variables. Les données avaient été encodées sous le logiciel Microsoft Excel 2013, l'analyse était faite à l'aide du logiciel IBM SPSS version 20.

### Considération éthique

Le protocole avait obtenu l'approbation du Comité d'éthique du Ministère Provincial de la Santé par son certificat numéro CNES 001/DPSK/129 PM/2019 du 20/04/2019. L'étude avait respecté les règles éthiques durant toute son exécution, par l'utilisation de l'anonymat durant toute l'enquête et l'obtention d'un consentement chez les enquêtées avant de commencer à poser des questions. Toutes les enquêtées ont adhéré librement sans être payées ou influencées par qui que ce soit.

## Résultats

Ici, nous présentons les résultats de 402 femmes qui ont répondu correctement à toutes les questions sur les 431 femmes enquêtées parmi lesquelles le taux d'utilisation de la contraception moderne était de 23,9% des cas.

### Caractéristiques sociodémographiques des femmes enquêtées

**Figure 1:**
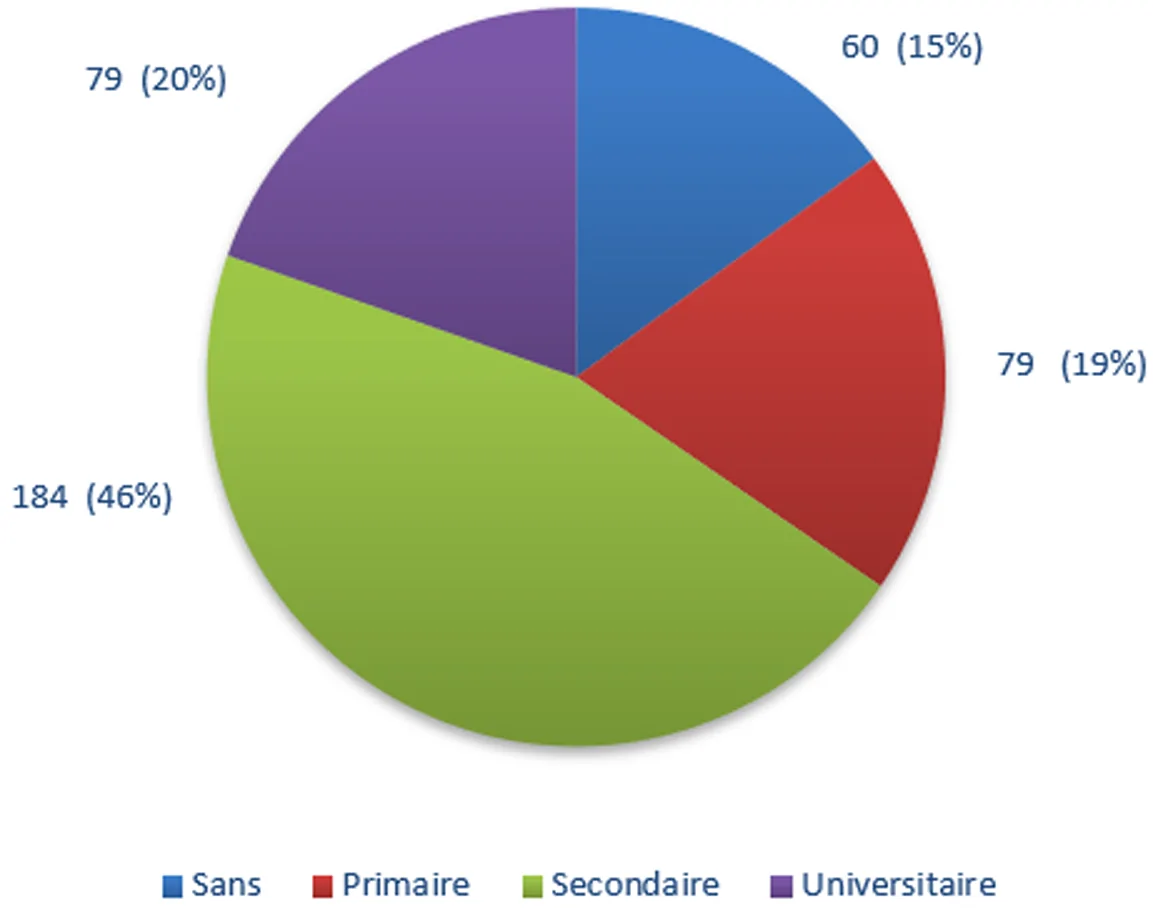
répartition des femmes enquêtées selon leur niveau d'étude

**Figure 2:**
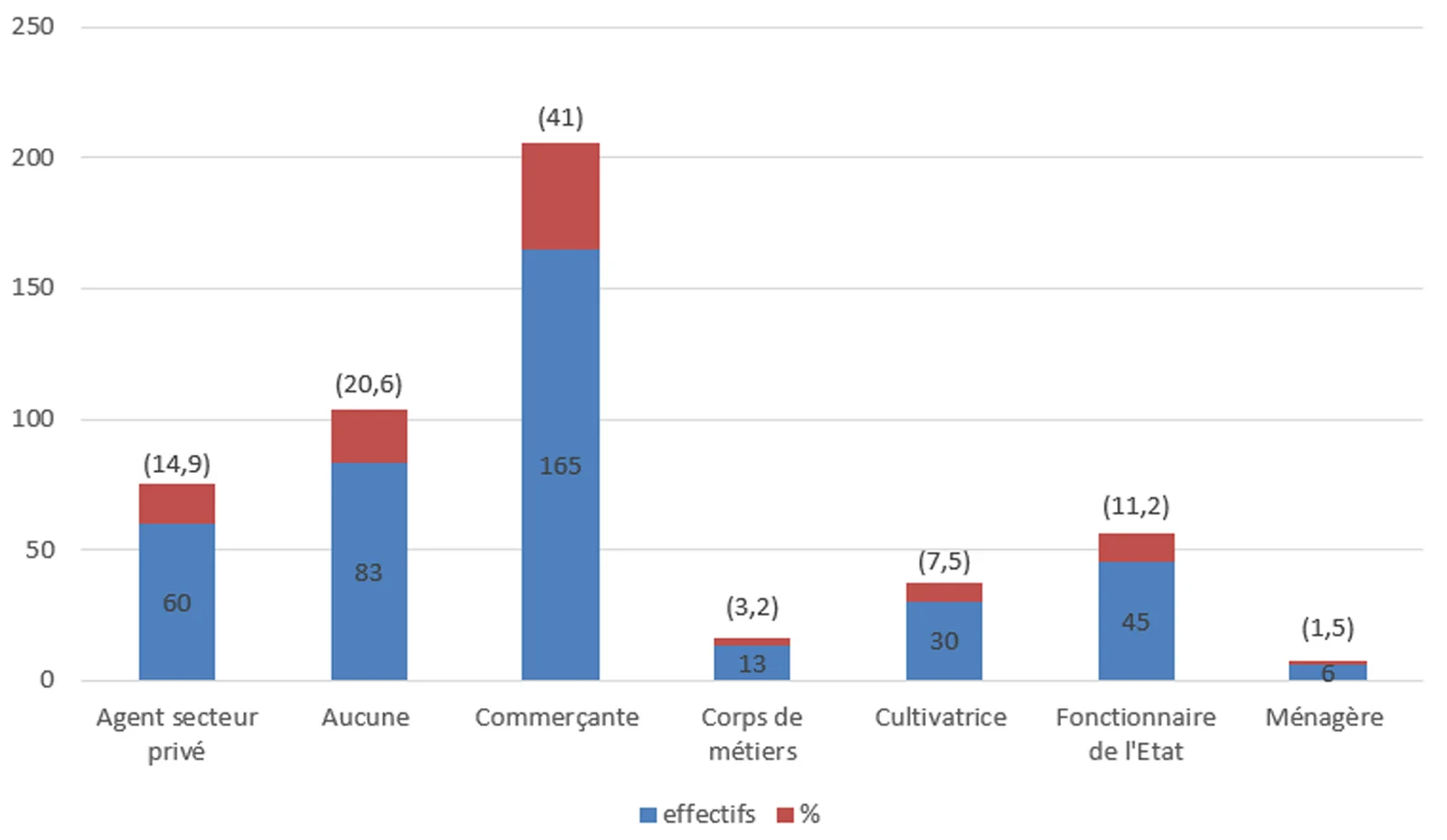
répartition des femmes enquêtées selon leur occupation

Les enquêtées avaient l'âge moyen de 31,97 ± 7,10 ans avec un minimum de 13 et un maximum de 45 ans. Nos enquêtées étaient pour la grande majorité (83,6%) mariées. Plus de la moitié (62,3%) avaient déjà perdu au moins un enfant (Tableau 1). Par rapport au niveau d'étude de nos enquêtées, moins de la moitié (45,8%) avaient un niveau d'étude secondaire et quelques-unes (19,7%) avaient le niveau primaire et universitaire (Figure 1). Pour ce qui concerne l'occupation (emploi), les commerçantes étaient majoritaires (41%) avec un bon nombre (20,6%) de femmes sans occupation (Figure 2). Notre étude montre une moyenne de 4,77 ± 2,39 enfants pour la parité, avec un minimum d'un enfant et un maximum de 11 enfants. La moyenne d'enfants souhaités par femme était de 6,60 ± 2,24 enfants avec un revenu mensuel moyen par ménage de 134 759 ± 207 465 FC, soit environ 81,6$/ménage.

**Tableau 1:** caractéristiques sociodémographiques des femmes enquêtées

Variables	% (n=402)
**Age des enquêtées (**31,97±7,10**) ans**	
≤19	4,0
20-29	36,3
30-39	37,6
≥40	22,1
**Statut matrimonial**	
Mariée	83,6
Séparée	8,5
Célibataire	6,2
Veuve	1,7
**Age de premier rapport sexuel (**18,96±3,10**) ans**	
≤14	6,5
15-19	51,5
20-24	34,6
≥25	7,5
**Espace intergénésique**	
Moins de 2 ans	53,0
2 ans et plus	47,0
**Désir d'avoir un enfant**	
Oui	80,4
Non	19,6
**Préférence en matière de sexe des enfants**	
Oui	64,7
Non	35,3
**Perte d'enfant**	
Oui	62,3
Non	37,7

### Attitudes des femmes de la Zone de santé d'Ibanda envers la contraception

Les paramètres retenus par rapport aux attitudes des femmes sur la contraception sont représentés ci-dessous (Tableau 2). Le constat était que plus de la moitié (57,5%) des femmes enquêtées avaient des attitudes défavorables à l'égard de la contraception. Concernant le lieu de procuration des contraceptifs, 45,7% des répondants se les procuraient à la pharmacie (Tableau 2).

**Tableau 2:** répartition des enquêtées selon leurs attitudes envers la contraception

Variables	% (n=402)
**Attitudes envers la contraception**	
Défavorable	57,5
Favorable	42,5
**Désir d'espacer les naissances**	
Non	55,2
Oui	44,8
**Désir de limiter les naissances**	
Non	85,1
Oui	14,9
**Participation aux séances de la planification familiale**	
Non	60,0
Oui	40,0
**Le mari approuve de PF**	
Non	69,9
Oui	30,1
**Dialogue conjugal sur la PF**	
Non	57,5
Oui	42,5
**Lieu de procuration des contraceptions**	
A la pharmacie	45,7
Au centre de santé	33,3
HGR	21,0

### Utilisation des méthodes contraceptives modernes par les femmes de la Zone de santé d'Ibanda

Dans la zone d'Ibanda, les types contraceptifs utilisés étaient successivement les contraceptifs injectables (40,6%), les pilules (33,3%), les préservatifs (16,6%) et le Jadelle (5,1%). Les raisons de non-utilisation des méthodes contraceptives pour certaines femmes étaient entre autres la peur des effets secondaires (33,1%), le désir d'avoir un enfant (27,4%), l'opposition du mari (23,8%), les croyances religieuses (11,8%) et la culture pronataliste.

### Facteurs explicatifs de l'inutilisation des méthodes contraceptives dans la Zone de santé d'Ibanda

Partant des caractères sociodémographiques de nos enquêtées, le niveau d'étude (OR=2,32; IC 95%: 1,00-5,47; p = 0,027) et l'occupation (profession) dont OR = 2,14; IC à 95%: 1,88-5,21, p = 0,017; sont les seuls caractères associés à l'utilisation des méthodes contraceptives. Cependant, nos résultats montrent que la perte d'enfants a fait en sorte que les femmes n'adhèrent pas à la contraception (OR: 0,07; IC 95%: 0,03-0,18; p = 0,000) (Tableau 3, Tableau 3.1). Quant aux variables liées aux connaissances des enquêtées sur la planification familiale (PF), nos résultats montrent que le fait de n'avoir jamais déjà entendu parler de la planification familiale (OR = 29,76; IC 95%: 4,39-58,86, p = 0,000 ), le fait de ne pas connaitre les avantages de la planification familiale (OR = 3,68; IC 95%: 1,93-7,12, p = 0,000), les contraceptifs (OR = 44,76; IC 95%: 6,63-87,18, p = 0,000), le fait de n'avoir jamais entendu parler des effets secondaires de la planification familiale (OR = 6,08; IC 95%: 2,60-14,91; p = 0,000) étaient significativement associés à l'utilisation des méthodes contraceptives modernes.

**Tableau 3:** relation entre les caractéristiques sociodémographiques et l'utilisation des méthodes contraceptives modernes

Variables	Utilisation des méthodes contraceptives		OR (IC 95%)	P
	Non (n=306)	Oui (n=96)		
**Age des enquêtées (ans)**				
≤19	14 (87,5)	2 (12,5)	1 (Réf)	
20-29	114 (78,1)	32 (21,9)	1,96 (0,39-13,23)	
30-39	114 (75,5)	37 (24,5)	2,27 (0,46-15,20)	
≥40	64 (71,9)	25 (28,1)	2,73 (0,53-18,81)	0,503
**Statut matrimonial**				
Célibataire	15 (60,0)	10 (40,0)	1 (Réf)	
Marié	264 (78,6)	72(21,4)	0,41 (0,16-1,03)	
Séparé	21 (61,8)	14 (38,2)	1,00 (0,31-3,25)	
Veuve	6 (85,7)	1 (14,3)	0,25 (0,01-2,79)	0,031
**Niveau d'étude des femmes**				
Sans	48 (80,0)	12 (20,0)	1 (Réf)	
Primaire	61 (77,2)	18 (22,8)	1,18 (0,48-2,91)	
Secondaire	147 (79,9)	37 (20,1)	1,01 (0,46-2,23)	
Universitaire	50 (63,3)	29 (36,7)	2,32 (1,00-5,47)	0,027
**Occupation**				
Aucune	66 (79,5)	17 (20,5)	1 (Réf)	
Agent secteur privé	39 (65,0)	21 (35,0)	2,09 (0,92-4,75)	
Commerçante	129 (78,2)	36 (21,8)	1,87(1,54-2,18)	
Corps de métiers	9 (69,2)	4 (30,8)	1,73 (0,39-7,25)	
Cultivatrice	28 (93,3)	2 (6,7)	0,28 (0,04-1,38)	
Fonctionnaire de l'Etat	29 (64,4)	16 (35,6)	2,14 (1,88-5,21)	0,017
Ménagère	6 (100)	0 (0,0)		
**Espace intergénésique**				
Moins de 2 ans	146 (77,2)	43 (22,8)	1 (Réf)	
2 ans et plus	160 (75,1)	53 (24,9)	1,12 (0,69-1,83)	0 ,617

**Tableau 3.1:** relation entre les caractéristiques sociodémographiques et l'utilisation des méthodes contraceptives modernes

Variables	Utilisation des méthodes contraceptives		OR (IC 95%)	P
	Non (n=306)	Oui (n=96)		
**Age du premier rapport sexuel (ans)**				
≤14	19 (73,1)	7 (26,9)	1 (Réf)	
15-19	152 (73,4)	55 (26,6)	0,90 (0,36-2,74)	
20-24	110 (79,1)	29 (20,9)	0,72 (0,25-2,09)	
25-29	25 (83,3)	5 (16,7)	0,54 (0,12-2,33)	0,473
**Désir d'un enfant**				
Non	61 (77,2)	18 (22,8)	1 (Réf)	
Oui	245 (75,8)	78 (24,2)	1,08 (0,58-2,02)	0,790
**Préférence en matière de sexe des enfants**				
Oui	200 (76,9)	60 (23,1)	1 (Réf)	
Non	106 (74,6)	36 (25,4)	1,13 (0,68-1,87)	0,609
**Perte d'enfant**				
Oui	161 (63,8)	91 (36,2)	1 (Réf)	
Non	145 (96,6)	5 (3,4)	**0,07 (0,03-0,18)**	**0,000**

L'analyse multivariée a montré que le statut matrimonial, la profession de la femme, la perte d'un enfant, le lieu de procuration de contraceptions, le désir d'espacer les naissances, le fait de connaître un contraceptif, les attitudes des femmes sur la contraception et le fait que le mari approuve la contraception sont significativement associés à l'utilisation des méthodes contraceptives modernes (p-value < 0,05) (Tableau 4).

**Tableau 4:** facteurs associés à l'utilisation des méthodes contraceptives chez les femmes enquêtées

Variables	Utilisation des méthodes contraceptives modernes				
Variables indépendantes	B	S.E.	Wald	ddl	P-value
Statut matrimonial	-0,738	0,423	3,051	1	0,081
Niveau d'étude	0,162	0,486	0,111	1	0,739
Profession des femmes	5,341	0,232	1,039	1	0,008
Perte d'enfants	2,609	1,146	5,184	1	0,023
Désir d'avoir un enfant	-0,611	0,462	1,751	1	0,186
Entendu parler de la PF	1,427	1,466	0,948	1	0,330
Connaissance des avantages de la PF	-0,005	0,520	0,000	1	0,993
Entendu des effets secondaires	0,475	0,608	0,611	1	0,434
Attitude envers la contraception	1,111	0,468	5,634	1	0,018
Désir d'espacer les naissances	1,041	0,502	4,300	1	0,038
Lieu de procuration de contraceptifs	2,215	0,394	3,330	1	0,005
Participation aux séances de PF	0,261	0,379	0,473	1	0,492
Le mari approuve la PF	1,992	0,433	21,113	1	0,000
Dialogue conjugal sur la PF	-0,016	0,457	0,001	1	0,972

LR: 245,27; Pseudo R^2^ de Nagelkerke: 0,58; Prob > chi2: 0,000; Nombre d'observation: 402

## Discussion

Cette étude s'était fixée comme objectif de rechercher les déterminants de l'inutilisation de la contraception moderne dans un milieu post-conflit. Une couverture de 23,9% pour la contraception moderne a été retrouvée. Le profil des femmes enquêtées avait une moyenne d'âge de 31,97±7,10 ans, mariées dans 83,6% des cas. Les raisons de non-utilisation de la contraception moderne étaient la peur des effets secondaires (33,1%), le désir d'avoir un enfant (27,4%), l'opposition du mari (23,8%), les croyances religieuses (11,8%), la culture pronataliste (1,6%). Certaines femmes utilisaient les pilules ou les injectables et s'approvisionnaient dans les pharmacies. Elles avaient manifesté la préférence en matière de sexe des enfants. Nous avons noté aussi que 62,3% des enquêtées avaient déjà perdu des enfants. L'indice de fécondité pour la femme congolaise étant de 6,6, le fait de perdre un enfant ou d'avoir une parité inférieure à 6 faisait en sorte qu'elles n'adhèrent pas à une contraception.

En comparant nos résultats à ceux trouvés au Gabon, l'on constate que les femmes en âge de procréer ont très fréquemment recours à une méthode contraceptive; mais il s'agit le plus souvent de méthodes naturelles qui sont peu efficaces [11]. Les raisons relèvent du préjugé ou de la mauvaise information: la peur de la stérilité (30,8% des réponses). Les jeunes femmes gabonaises découvraient à la fois les vrais effets secondaires et les mythes à partir de leurs réseaux sociaux [11]. Cette influence négative des effets secondaires sur l'utilisation de la contraception moderne a été signalée dans notre étude.

Le Nigéria fait partie des quelques pays subsahariens d'Afrique avec une utilisation contraceptive constamment faible de 15% chez les femmes. Les résultats d'une étude menée dans ce pays en 2016 par Salami *et al*. [12], montrent que certains facteurs de fond des femmes comme l'état de résidence, l'éducation, l'indice de richesse et le nombre d'autres épouses ont une relation directe et indirecte avec l'usage de la contraception. La probabilité qu'une femme utilise une méthode contraceptive augmente considérablement avec le niveau de scolarité, le nombre d'enfants vivants, et varie significativement selon l'état de résidence, le nombre d'autres épouses et la préférence pour la fertilité. Au Ghana en 2014, Adongo *et al*. [13] montrent que les besoins non satisfaits en matière de contraception sont omniprésents aussi bien au nord qu'au sud de ce pays. Les causes possibles relevées par cette étude sont nombreuses, notamment l'utilisation du préservatif, qui était généralement perçue comme inhibant l'érection et donc capable d'induire l'impuissance masculine. Ils insistent aussi sur la place des craintes et des idées fausses dominantes dans l'inutilisation de la contraception moderne, qui peut être améliorée par la communication. Dans le même pays, Eliason *et al*. [14], trouvent que le manque d'éducation formelle des femmes, les croyances socioculturelles, la communication entre conjoints et les heures d'ouverture favorables ainsi que la distance à parcourir jusqu'à l'établissement de santé étaient notés comme facteurs influençant l'utilisation de contraceptifs modernes. Ces résultats ne sont pas loin de ceux trouvés dans notre travail, faisant penser aux mêmes réalités dans les pays à faibles revenus et particulièrement ceux d'Afrique.

Nos résultats sont également comparables à ceux trouvés en Ethiopie par Alemayehu *et al*. [15]. Dans ce pays de la Corne de l'Afrique, la majorité (64%) des femmes mariées en âge de procréation connaissent les *Long-Acting and Permanent Methods of contraception* (LAPM). Plus de la moitié (53,6%) des enquêtées avaient une attitude négative envers la pratique des LAPM, pendant que la prévalence globale de l'utilisation des LAPM était de 12,3%. Le fait d'avoir des connaissances élevées était associé à l'utilisation des LAPM (OR = 7,9, IC 95%: 3,1-18,3). Les mères qui ont eu deux grossesses ou plus étaient 3 fois plus susceptibles d'utiliser LAPM (OR = 2,7, IC 95% : 1,4-5,1) [15].

Gebremariam *et al*. [16] en 2014 en Ethiopie trouvent que la principale source d'approvisionnement des contraceptifs était les centres de santé publics (71,3%) et l'hôpital (20,8%) contrairement à notre étude où c'était dans les pharmacies. Les principales raisons d'usage des contraceptifs étaient la peur de tomber enceinte (38,9%) et d'allaiter exclusivement (25%). Le niveau de scolarité des couples, le désir d'avoir un enfant dans les deux ans ou la planification familiale avec un partenaire et l'utilisation de contraceptifs modernes ont été les facteurs déterminant l'utilisation de contraceptifs [15,16]. Des résultats similaires aux nôtres avaient été trouvés au Kenya [17]. Néanmoins, contrairement à notre étude, les principales sources d'approvisionnement en méthodes contraceptives au Kenya et en Ethiopie étaient les centres de santé publics et l'hôpital respectivement [16,17]. Ce qui montre une implication de l'Etat dans la promotion des méthodes contraceptives dans certains pays d'Afrique subsaharienne.

Le Rwanda est un pays qui a connu une augmentation spectaculaire de l'utilisation de la contraception cette dernière décennie, entraînant une baisse importante du taux de fécondité, alors que vers les années 1983, ce pays connaissait un taux de fécondité très élevé allant jusqu'à 8,5 enfants par femme en moyenne. Entre 2005 et 2010, le Rwanda a connu une des chutes les plus rapides qui aient été observées dans l'histoire des enquêtes démographiques et de santé (EDS), à un rythme de 25% [18]. Ces changements peuvent être attribués au leadership et à l'engagement renouvelé du gouvernement rwandais en faveur de la planification familiale. Un exemple à suivre par d'autres gouvernements africains pour espérer voir une hausse du taux d'utilisation de la contraception moderne dans tous les pays d'Afrique subsaharienne.

Des faibles prévalences ont été rapportées dans plusieurs études en République démocratique du Congo variant entre 15% et 30% [10,19-21]. Une étude répond à la question: avec un accès physique raisonnable et des besoins non satisfaits relativement élevés, pourquoi la prévalence contraceptive moderne est-elle si faible? Cinq principaux obstacles sont ressortis des discussions de groupe: la peur des effets secondaires (en particulier la stérilité), les coûts de la méthode, les normes socioculturelles (en particulier la position dominante de l'homme dans la prise de décision familiale), la pression des membres de la famille pour éviter la contraception moderne, et le manque d'information/de désinformation [22].

Ntambue *et al*. avaient réalisé une étude en 2015 dont l'objectif était de déterminer la prévalence contraceptive moderne et les barrières à l'utilisation des méthodes contraceptives modernes. Dans leur étude, les auteurs avaient trouvé que la prévalence contraceptive moderne à Dibindi était de 18,4% [7]. Le refus d'utiliser les méthodes contraceptives modernes était lié au désir de maternité, à l'interdiction religieuse, à l'opposition du conjoint et à la crainte des effets secondaires [23]. Le niveau de scolarité des couples, le désir d'avoir d'autres enfants, le manque d'emploi ou d'activité rentable, le manque de dialogue avec le partenaire, la désapprobation du conjoint, les croyances religieuses, la crainte des effets secondaires, le niveau de vie (bas et moyen) et l'ignorance des avantages de la contraception sont autant de facteurs rapportés dans différentes études en RDC [10,19,23-25]. Ceci suggère d'étudier à fond la question de l'organisation des services de PF dans les pays en général et dans les zones de santé en particulier. Au lieu de centrer les services sur la femme seule, il est impérieux d'étudier comment intégrer le couple, de renforcer les méthodes de sensibilisation dans les communautés, de jouer beaucoup sur l'autonomisation de la femme par des activités génératrices de revenus, de faire intervenir les leaders dans la PF. Bien d'autres actions sont envisageables pour améliorer la prévalence de la contraception moderne en RDC et dans la Zone de santé d'Ibanda.

## Conclusion

Ce travail a relevé une prévalence contraceptive moderne de 23,9% dans une zone post-conflit. Les femmes de cette Zone de santé connaissent la contraception moderne, mais ne l'utilisent pas. Plusieurs facteurs limitent ces femmes à l'utiliser; le manque de profession, la perte de l'enfant dans le foyer, le lieu de procuration de contraceptifs, le manque de désir d'espacer les naissances, le fait de méconnaître un contraceptif, les attitudes négatives des femmes sur la contraception et le fait que le mari n'approuve pas la contraception dans le foyer. Ainsi, l'autonomisation de la femme, le renforcement de la sensibilisation étendue à l'homme et l'amélioration des services des PF dans les structures sanitaires amélioreraient l'utilisation des contraceptifs modernes dans la Zone de santé d'Ibanda.

### Etat des connaissances sur le sujet

- La planification familiale reste l'intervention primordiale pour réduire la mortalité maternelle et infantile;
- Les pays à faible revenu présentent une prévalence contraceptive très faible au monde;
- Les services de planification ne sont pas efficaces là où les femmes en ont besoin.

### Contribution de notre étude à la connaissance

- La prévalence contraceptive moderne est faible dans des zones à post-conflit, cas de la Zone de santé d'Ibanda, dans la ville de Bukavu à l'est de la République démocratique du Congo;
- Les facteurs qui limitent l'utilisation de la contraception moderne par les femmes en zone post-conflit ne sont pas différents de ceux évoqués par les autres femmes d'Afrique;
- En RDC, le programme de planification familiale doit s'améliorer et s'étendre au besoin à l'homme dans chaque couple avec un engagement fort de l'État pour pouvoir inverser la tendance.

## Remerciements

Nous remercions le comité de gestion de l’Université Evangélique en Afrique (UEA) pour l’appui financier de ce travail. Nous remercions également la Zone de santé d’Ibanda d'avoir facilité aux enquêteurs la collecte des données.

## References

[1] Lopez-Del Burgo C, de Irala J (2016). Modern contraceptive methods: a new misleading definition. Contraception.

[2] Gueye AS, Ndiaye P, Tal Dia A, Kessler C, Fergusson A (2004). Fertilité, mortalité maternelle et survie des enfants du district sanitaire de Kolda (Sénégal). Dakar Med.

[3] Leyé MM, Faye A, Diongue M, Wone I, Seck I, Ndiaye P (2015). Déterminants de l'utilisation de la contraception moderne dans le district sanitaire de Mbacké (Sénégal). Sante Publique.

[4] Gomard M (2017). Facteurs influençant l'utilisation de la contraception à Mayotte. Enquête auprès des femmes consultant en dispensaire (Doctoral dissertation. Université Toulouse lll-Paul Sabatier).

[5] Mariko S, Ayad M, Hong R, Kéïta O, Diop M Pratique contraceptive et importance des besoins non satisfaits en matière de planification familiale au Mali, de 1995 à 2006: analyses approfondies des enquêtes démographiques et de santé au Mali, 1995-1996 2001 et 2006. République du Mali Septembre 2009.

[6] Fassassi R (2007). Les facteurs de la contraception en Côte d'Ivoire au tournant du siècle. Les collections du CEPED Collection Regards sur, CEPED/GRIPPS. Paris. ENSEA.

[7] Ntambue AM, Tshiala RN, Malonga FK, Ilunga TM, Kamonayi JM, Kazadi ST (2017). Utilisation des méthodes contraceptives modernes en République Démocratique du Congo: prévalence et barrières dans la zone de santé de Dibindi à Mbuji-Mayi. Pan Afr Med J.

[8] Morrisson C (2002). SANTÉ ÉDUCATION ET RÉDUCTION DE LA PAUVRETÉ. Centre de Développement de l'OCDE. Cahier de Politique Économique N°19.

[9] UNFPA-RD Bulletin n°1 de la surveillance des décès maternels et riposte (SDMR) janvier-juin 2018 en République Démocratique du Congo. Bulletin n°1 SDMR Janvier-Juin.

[10] Matungulu CM, Kandolo SI, Mukengeshayi AN, Nkola AM, Mpoyi DI, Mumba SK (2015). Déterminants de l'utilisation des méthodes contraceptives dans la zone de santé Mumbunda à Lubumbashi. République Démocratique du Congo. Pan Afr Med J.

[11] Chiesa-Moutandou S, Wantou GT (2001). Le comportement contraceptif des Gabonaises les méthodes modernes: Faible taux d'utilisation et déficit d'information. Médecine d'Afrique Noire.

[12] Salami IC, Oladosu M Socio-Demographic, Factors. Contraceptive Use and Fertility Preference among Married Women in SouthSouth Region of Nigeria. In 3rd International Conference on African Development Issues ISSN 2016.

[13] Adongo PB, Tabong PT, Azongo TB, Phillips JF, Sheff MC, Stone AE (2014). A comparative qualitative study of misconceptions associated with contraceptive use in southern and northern ghana. Front Public Health.

[14] Eliason S, Awoonor-Williams JK, Eliason C, Novignon J, Nonvignon J, Aikins M (2014). Determinants of modern family planning use among women of reproductive age in the Nkwanta district of Ghana: a case-control study. Reprod Health.

[15] Alemayehu M, Belachew T, Tilahun T (2012). Factors associated with utilization of long acting and permanent contraceptive methods among married women of reproductive age in Mekelle town. Tigray region, north Ethiopia. BMC Pregnancy Childbirth.

[16] Gebremariam A, Addissie A (2014). Modern contraceptive method mix and factors affecting utilization of modern contraceptives among married women in Adigrat town, Tigray, Northern Ethiopia. Fam Med Med Sci Res.

[17] Ochako R, Mbondo M, Aloo S, Kaimenyi S, Thompson R, Temmerman M (2015). Barriers to modern contraceptive methods uptake among young women in Kenya: a qualitative study. BMC Public Health.

[18] Belohlav KA, Nolan L (2013). Besoins non Satisfaits en Planification Familiale et Demandes pour des Familles plus petites au Rwanda: 4.

[19] Kadima CL, Nyembo EM, Muleba FK, Katenga GB Female Use Of Artificial Contraceptive Methods In Mbuji-Mayi: Prevalence And Some Determinants. Int J Recent Sci Res.

[20] Mbarambara PM, Kingombe CZ, Mututa PM, Bisangamo CK (2016). Déterminants de l'utilisation des contraceptifs par les femmes à l'Hôpital Général de Référence de Bagira, en République Démocratique du Congo. International Journal of Innovation and Applied Studies.

[21] Mbarambara PM, Mumbilyia E, Mututa PM, Ndage AM (2016). Niveau d'acceptabilité de la planification familiale dans la Zone de Santé de Kadutu à l'Est de la RD Congo. International Journal of Innovation and Applied Studies.

[22] Kayembe PK, Fatuma AB, Mapatano MA, Mambu T (2006). Prevalence and determinants of the use of modern contraceptive methods in Kinshasa, Democratic Republic of Congo. Contraception.

[23] Kwete D, Binanga A, Mukaba T, Nemuandjare T, Mbadu MF, Kyungu MT (2018). Family Planning in the Democratic Republic of the Congo: Encouraging Momentum, Formidable Challenges. Glob Health Sci Pract.

[24] Mbeva JB, Karemere H, Prudence MN, Nyavanda L, Mundama JP (2018). Facteurs explicatifs des déces maternels en milieu hospitalier: une étude au niveau de six zones de sante dans l'est de la République Démocratique du Congo. International Journal of Innovation and Applied Studies.

[25] Muanda M, Gahungu Ndongo P, Taub LD, Bertrand JT (2016). Barriers to Modern Contraceptive Use in Kinshasa, DRC. PLoS One.

